# Development and Validation of an Antigen-Capture ELISA for Quantitative Detection of Donkey-Origin Rotavirus: Application in Vaccine Manufacturing Process Monitoring

**DOI:** 10.3390/microorganisms14091874

**Published:** 2026-08-24

**Authors:** Zenan Zhang, Xing Guo, Kui Guo, Wei Guo, Cheng Du, Yan Yang, Wenjing Dong, Xiaoyu Chu, Yuezhi Lin, Xiaojun Wang

**Affiliations:** 1 State Key Laboratory for Animal Disease Control and Prevention, Harbin Veterinary Research Institute, The Chinese Academy of Agricultural Sciences, Harbin 150069, China; zhangzenan1995@163.com (Z.Z.); guoxing@caas.cn (X.G.); guokuiking@163.com (K.G.); guowei@caas.cn (W.G.); ducheng@caas.cn (C.D.); m13467961834@163.com (Y.Y.); dw2235784355@163.com (W.D.); chuxiaoyu613@163.com (X.C.); linyuezhi@caas.cn (Y.L.); 2 Institute of Western Agriculture, The Chinese Academy of Agricultural Sciences, Changji 831100, China

**Keywords:** donkey rotavirus, acELISA, VP6, vaccine manufacturing, tangential flow filtration

## Abstract

Rotavirus A (RVA) causes severe diarrhea in donkey foals, but rapid quantitative antigen assays for vaccine development and manufacturing control are lacking. We developed a VP6-based antigen-capture ELISA (acELISA) for donkey-origin RVA using rabbit anti-RVA polyclonal IgG as the capture antibody and horseradish peroxidase-conjugated mouse anti-VP6 monoclonal antibody 3F6 as the detector antibody. Assay conditions were optimized, and performance was evaluated through standard-curve analysis, specificity and repeatability testing, Western blot (WB) comparison, median tissue culture infectious dose (TCID_50_), reverse transcription quantitative polymerase chain reaction (RT-qPCR), ultrafiltration membrane screening, and pilot-scale tangential flow filtration (TFF) monitoring. The optimized acELISA detected VP6 at 1 ng/mL and showed a linear range of 1–25 ng/mL, with no cross-reactivity with eight common equine pathogens, and intra- and inter-assay coefficients of variation below 10%. The acELISA estimates were consistent with Western blot densitometry. In process applications, the assay supported the selection of a 100 kDa membrane and monitored pilot-scale TFF, yielding 75.69% VP6 recovery and an 18-fold increase in antigen purity. This acELISA provides a specific and practical in-process tool for quantitative antigen monitoring in donkey rotavirus vaccine production and is intended to complement, rather than replace, infectivity- and genome-based assays.

## 1. Introduction

Rotavirus (RV) is a major enteric pathogen that causes acute gastroenteritis in young animals and humans. In equids, rotavirus-associated diarrhea in foals can lead to dehydration, growth retardation, veterinary costs, and occasionally death [1,2]. Rotaviruses are non-enveloped viruses of the genus *Rotavirus*, family *Sedoreoviridae* (formerly *Reoviridae*) [3,4]. The genome consists of 11 segments of double-stranded RNA with a total size of approximately 18.5 kb, encoding six structural proteins (VP1–VP4, VP6 and VP7) and six non-structural proteins (NSP1–NSP6). The mature virion contains an inner capsid, a VP6-rich intermediate capsid, and an outer capsid formed mainly by VP4 and VP7 [3,5]. VP6, which is encoded by genome segment 6, is 397 amino acids long, has a predicted molecular mass of approximately 45 kDa, and assembles into highly stable homotrimers that form the 260 trimeric spikes of the intermediate capsid layer. It is the most abundant structural protein of the virion (~51% of total virion protein). It is strongly immunogenic and carries the group (species)- and subgroup-specific antigenic determinants that define RVA. Its trimeric surface epitopes are conformation-dependent, which is directly relevant to the choice of detector antibody in antigen-capture assays [6]. The VP7 and VP4 proteins define the G and P genotypes, respectively. RVA is isolated from approximately 20–60% of diarrhea cases on breeding farms, and morbidity within an affected foal crop may exceed 50% during outbreaks. Case fatality is usually low (<5%) with prompt supportive care, but rises sharply in neonates infected during the first weeks of life [1,2]. Globally, G3P[12] and G14P[12] genotypes account for the large majority of equine RVA infections and are included in current equine vaccines. In China, donkey-origin G3P[12] strains have been described [7,8,9]. Emerging or reassortant genotypes are increasingly reported and are not necessarily covered by these vaccines [2], creating a continuing need for improved prevention and process-controlled vaccine development.

Reliable antigen quantification is essential for vaccine process development and batch consistency [10,11]. Infectivity assays such as median tissue culture infectious dose (TCID_50_) are valuable for live virus harvests but are slow, operator-dependent, and unsuitable once the virus has been inactivated. Reverse transcription quantitative polymerase chain reaction (RT-qPCR) is sensitive and high-throughput, but it measures genome copies rather than antigenically intact particles and can detect free or degraded nucleic acid [12,13]. VP6 is the most abundant rotavirus structural protein and is relatively conserved within group A rotaviruses, making it an attractive target for antigen detection and vaccine quality control [14,15,16]. Previous VP6-based ELISAs have supported rotavirus diagnostics and vaccine quality assessment in other hosts, but a validated quantitative assay for donkey-origin RVA process monitoring has not been reported [17,18,19]. Accordingly, several VP6-based immunoassays have been reported. Lee et al. reported a cell-culture-coupled immunochromatographic assay targeting the VP6 antigen of group A human rotaviruses, which detected infectious virus at concentrations as low as 1.99 TCID_50_/mL [20]. Memon et al. reported a polyclonal antibody-based antigen-capture ELISA for porcine rotavirus with a detection limit of 50 ng/mL of VP6 protein [21]. Despite this, several VP6-based sandwich ELISAs have previously been reported for human and porcine rotaviruses and have been applied mainly for diagnostic purposes or quality control during vaccine production. Most of these assays employ monoclonal or polyclonal antibodies directed against VP6 and provide qualitative or semi-quantitative detection of rotavirus antigen. However, these assays were developed for other host species and have not been validated for donkey-origin Rotavirus A or evaluated as process analytical tools for monitoring antigen recovery during vaccine manufacturing. Therefore, there remains a need for a quantitative assay specifically optimized for donkey-origin RVA and suitable for in-process monitoring during vaccine production.

Although VP6 is relatively conserved among group A rotaviruses, assay performance depends on the affinity and epitope recognition of the antibody pair used. Existing VP6 antigen-capture ELISAs were developed using human or porcine rotavirus strains and have not been validated for donkey-origin isolates. Furthermore, the recent emergence of novel donkey RVA genotypes, including G3P[59], highlights the need for assays specifically evaluated using donkey vaccine strains. In vaccine manufacturing, quantitative antigen determination must be validated for the target production strain to ensure accurate monitoring of antigen recovery, process consistency, and batch-to-batch quality.

Downstream vaccine manufacturing increasingly uses tangential flow filtration (TFF) because it is scalable and can combine concentration with diafiltration under relatively mild conditions. For whole-virus or virus-like-particle vaccines, TFF optimization requires an assay that can rapidly measure antigen retention, product recovery, and impurity clearance at multiple processing steps [22,23,24]. In this study, we developed and validated a VP6-targeted antigen-capture ELISA using rabbit anti-RVA polyclonal IgG and HRP-conjugated mouse anti-VP6 mAb 3F6. We then compared the assay with Western blot (WB) densitometry, TCID_50_, and RT-qPCR and applied it to ultrafiltration cutoff selection and pilot-scale TFF monitoring for donkey-origin RVA vaccine manufacturing.

## 2. Materials and Methods

### 2.1. Animals

The 6–8-week-old New Zealand white rabbits and 6-week-old female BALB/c mice used in this study were obtained from the Changsheng Biotechnology Company (Liaoning, China). All procedures were performed under anesthesia to minimize pain and distress, in accordance with the recommendations of the Ethics Committees of HVRI. The Animal Ethics Committee approval numbers are Heilongjiang-SYXK (Hei) 241122-06-GR (approval date: 22 November 2024) and 241126-05-GR (approval date: 26 November 2024).

### 2.2. Virus Strains, Cells, and Reagents

Two donkey-origin group A rotavirus strains were used in this study: SD2109 (G3P[12] genotype), originally isolated from a diarrheic donkey foal in Shandong Province, China, and HB2503(G3P[59] genotype), isolated from a diarrheic donkey foal in Hebei Province, China; both were maintained in our laboratory. Marc-145 cells (African green monkey kidney cell line) were cultured in Dulbecco’s modified Eagle’s medium (DMEM; Sigma-Aldrich, St. Louis, MO, USA) supplemented with 10% fetal bovine serum (FBS; PAA, Pasching, Austria; No. A-005N), 100 U/mL penicillin, and 100 μg/mL streptomycin at 37 °C in a humidified atmosphere containing 5% CO_2_. The prokaryotic expression vectors pET-32a (+) and competent cells DE3 (BL21) were maintained in our laboratory.

The following equine pathogens were obtained for cross-reactivity testing: Equine Infectious Anemia Virus (EIAV), Equine Influenza Virus (EIV), Equine Herpesvirus Type 1 (EHV-1), Equine Herpesvirus Type 4 (EHV-4), Equine Arteritis Virus (EAV), *Salmonella abortus equi* (*S. abortus equi*), *Escherichia coli* (*E. coli*), and *Trypanosoma evansi* (*T. evansi*). All pathogen preparations were collected and stored in our laboratory [25].

All work involving infectious rotavirus was performed in a biosafety cabinet (BSC) within a biosafety level 2 (BSL-2) laboratory, in accordance with the Regulations on the Biosafety Management of Pathogenic Microorganism Laboratories of the People’s Republic of China. All solid waste was decontaminated by autoclaving, and all liquid waste was chemically inactivated by alkaline treatment (NaOH solution) prior to disposal.

### 2.3. Virus Propagation and Purification

Virus stocks were activated with trypsin at 50 μg/mL and inoculated onto 90% confluent Marc-145 cells at an MOI of 0.01. The maintenance medium was serum-free DMEM supplemented with 10 μg/mL trypsin. Cultures were harvested when extensive cytopathic effect (CPE) was observed, typically at 36–48 h post-infection, and subjected to three freeze-thaw cycles [26]. After clarification at 8000× *g* for 30 min at 4 °C, SD2109 virions were pelleted by ultracentrifugation at 100,000× *g* for 2 h at 4 °C, resuspended in PBS, and stored at −80 °C. Virion morphology was examined by negative-staining transmission electron microscopy with 2% phosphotungstic acid [27,28]. Protein concentration was measured spectrophotometrically, adjusted to 2 mg/mL, and analyzed by sodium dodecyl sulfate polyacrylamide gel electrophoresis (SDS-PAGE).

### 2.4. Preparation and Purification of Rabbit Anti-Rotavirus Polyclonal Antibody

Purified SD2109 virions (obtained as described in Section 2.3) were used to immunize New Zealand White rabbits. The primary immunization contained 1 mg purified virion protein emulsified with Freund’s complete adjuvant; three booster immunizations were given at 2-week intervals using 1 mg antigen emulsified with Freund’s incomplete adjuvant. Serum was collected 14 days after each immunization and monitored by indirect ELISA against purified SD2109 virions and recombinant VP6 (rVP6). When titers exceeded 1:10,000, terminal blood collection was performed. IgG was purified by caprylic acid–ammonium sulfate precipitation, dialyzed against PBS, quantified spectrophotometrically, and evaluated by SDS-PAGE, WB, and an indirect immunofluorescence assay (IFA).

### 2.5. Cloning, Expression, and Purification of Recombinant VP6 Protein

The full-length VP6 open reading frame gene (ORF, 1194 bp) of the SD2109 strain was amplified using the PrimeScript™ One Step RT-PCR Kit Ver.2 (Dye Plus) (TaKaRa, Beijing, China; No. RR057A) according to the manufacturer’s instructions. The reaction was performed in a 50 µL volume containing 1 µL of PrimeScript 1 step Enzyme Mix, 25 µL of 2× 1 step Buffer, 1 µM of each primer, and 2 µL of viral RNA template. Reverse transcription was carried out at 50 °C for 30 min, followed by initial denaturation at 94 °C for 2 min, and 30 cycles of 94 °C for 30 s, 58 °C for 30 s, and 72 °C for 1 min, with a final extension at 72 °C for 5 min. The gene-specific primers (VP6-ORF-F: 5′-atggatgtcctgtactccttgtcaaaaact-3′, VP6-ORF-R: 5′-tcatttgacaagcatgcttctaatggaagc-3′) were designed to amplify the complete VP6 ORF from the start codon to the stop codon and were commercially synthesized. The amplification was verified by electrophoresis on a 1% agarose gel and was recovered with a gel extraction kit (TIANGEN, Beijing, China; No. DP209-02). The purified product was cloned into the prokaryotic expression vector pET-32a (+) through BamHI and HindIII restriction sites. This vector carries an N-terminal TrxA- 6xHis- S fusion tag, which facilitates soluble expression (~17.7 kDa in total, including the linker, Figure S2). The positive recombinant plasmid pET-32a-SD2109-VP6 was confirmed by bidirectional Sanger sequencing (Ruibio BioTech Co., Ltd., Harbin, China) and was transformed into *E. coli* BL21 (DE3) competent cells. When the bacterial culture reached an OD_600_ nm of approximately 0.6–0.8, isopropyl-β-D-thiogalactopyranoside (IPTG) was added to the medium at a final concentration of 0.5 mM, followed by induction at 16 °C, 25 °C, and 37 °C for 20 h to optimize protein expression conditions. The cultures were collected by centrifugation at 6000× *g* for 5 min at 4 °C, resuspended in PBS buffer (pH 7.4), and disrupted by sonication. The recombinant SD2109-VP6 protein (rVP6) was purified using High Affinity Ni-Charged Resin (GenScript, Nanjing, China; No. L00223-50) according to the manufacturer’s instructions. The eluted fractions were pooled, dialyzed against PBS, and the protein concentration was determined by spectrophotometry. The purity and identity of rVP6 were confirmed by SDS-PAGE and WB analysis using a mouse anti-His tag antibody, rabbit anti-rotavirus polyclonal serum, and clinical donkey anti-rotavirus positive serum.

### 2.6. Preparation and Characterization of Mouse Anti-VP6 Monoclonal Antibody

BALB/c mice were immunized intraperitoneally with purified rVP6 protein (100 μg/mouse) emulsified with Freund’s complete adjuvant for the primary immunization. Three booster immunizations were administered at 2-week intervals using 100 μg rVP6 emulsified with Freund’s incomplete adjuvant. Serum antibody titers were monitored by iELISA 10 days after the third immunization. The titer was confirmed to have reached a sufficient level (≥1:100,000), and the mouse received a final intraperitoneal boost of 100 μg rVP6 protein in PBS without adjuvant three days before fusion. The mouse was euthanized, and the spleen was removed aseptically. A single-cell suspension was prepared by repeatedly flushing the spleen with serum-free RPMI 1640 using a syringe, followed by filtration through a 70 μm cell strainer (Falcon^®^, Corning, NY, USA; No. 352350). Splenocytes and log-phase SP2/0 myeloma cells were mixed at a ratio of 5:1 and fused with a 50% (*w*/*v*) polyethylene glycol solution (PEG; Sigma-Aldrich, St. Louis, MO, USA; No. P7306) added dropwise over 1 min at 37 °C, followed by gradual dilution with serum-free RPMI 1640, essentially as described by Köhler and Milstein [29,30]. Fused cells were resuspended in RPMI 1640 containing 20% FBS and seeded into 96-well plates pre-coated with mouse peritoneal feeder cells. Hybridomas were selected in hypoxanthine-aminopterin-thymidine (HAT; Sigma-Aldrich, St. Louis, MO, USA; No.H0262) medium and screened by indirect ELISA using SD2109 strain purified virions-coated plates.

Positive hybridoma clones were further characterized by WB using denatured rVP6, dot blot using native rVP6, and IFA against rotavirus-infected Marc-145 cells. The lead clone, designated 3F6, which recognized a conformational epitope and exhibited strong reactivity in all characterization assays, was stabilized by two rounds of limiting dilution followed by one round of flow cytometric cell sorting (SONY, Tokyo, Japan), yielding a stable monoclonal cell line. The monoclonal antibody isotype was determined using a commercial mouse immunoglobulin isotyping kit (Southern Biotech, Birmingham, AL, USA; No. 5300-05). Large-scale antibody production was performed by the ascites method in pristane-primed BALB/c mice [30]. The monoclonal antibody was purified from ascites fluid by Protein A/G affinity chromatography and conjugated to horseradish peroxidase (HRP; Sigma-Aldrich, St. Louis, MO, USA; No. P6782) using the periodate oxidation method [25,30]. Antibody titers before and after conjugation were measured by ELISA.

### 2.7. Indirect ELISA (iELISA), WB, IFA

For iELISA, plates were coated with purified SD2109 virions or rVP6 at 1 μg/mL overnight at 4 °C and blocked with 5% skimmed milk in PBST for 2 h at 37 °C. Serially diluted serum or antibody was incubated for 2 h at 37 °C, followed by HRP-conjugated secondary antibody and 3,3′,5,5′-tetramethylbenzidine (TMB) substrate. Absorbance was measured at 450 nm. For WB, proteins were separated by 12% SDS-PAGE, transferred to nitrocellulose membranes, blocked with 5% skimmed milk in Tris-buffered saline with 0.1% Tween-20 (TBST), and incubated with rabbit polyclonal IgG, hybridoma supernatant, mAb 3F6, mAb 5A2, anti-His tag antibody, or donkey positive serum. DyLight800- or DyLight680-conjugated IgG secondary antibodies (1:5000–10,000 dilution, KPL, Gaithersburg, MA, USA; No. 5230-0415/Novus, Centennial, CO, USA; No. NB120-6700FR) were used, and bands were visualized with an Odyssey infrared imaging system (LI-COR, Lincoln, NE, UK). For IFA, Marc-145 cells infected with SD2109 were fixed with 4% paraformaldehyde and permeabilized with 0.1% Triton X-100 at 3–4 days post-infection. Cells were incubated with primary antibody, followed by fluorescein isothiocyanate (FITC)-conjugated secondary antibody (1:500 dilution; Invitrogen, Waltham, MA, USA; No. A11029) and 4′,6-diamidino-2-phenylindole (DAPI; Beyotime, Shanghai, China; No. C1006) nuclear staining.

### 2.8. Development and Optimization of Antigen-Capture ELISA (acELISA)

The acELISA was developed using purified rabbit anti-rotavirus polyclonal IgG as the capture antibody and HRP-conjugated mouse anti-VP6 monoclonal antibody (clone 3F6) as the detector antibody. The optimal working concentrations of both antibodies were determined by checkerboard titration. Briefly, 96-well microplates (Corning, NY, USA; No. 3595) were coated with serial dilutions of rabbit polyclonal IgG (1000/400/200/100 ng/well) in coating buffer. A fixed concentration of rVP6 protein was added after blocking, followed by serial dilutions of HRP-conjugated mAb 3F6 (1000–6000 dilution HRP-mAb 3F6). The combination yielding the highest positive-to-negative (P/N) ratio was selected.

Subsequent optimization experiments were performed to determine the following parameters: (i) blocking agent—PBS containing 5% skim milk (BD-Pharmingen; San Diego, CA, USA; No. 232100), PBS containing 1% bovine serum albumin (BSA; Biosharp, Hefei, China; No. BS114), commercial microplate stabilizer I, and commercial microplate stabilizer II (Yingchuang, Huzhou, China; No. IBS-001/002), with blocking conditions evaluated at 37 °C for 2 h; (ii) sample diluent buffer—PBS, PBS containing 0.5% Triton X-100, and 0.25 M EDTA buffer; (iii) HRP-conjugated antibody diluent buffer—PBS containing 0.05% Tween-20, commercial HRP stabilizer I, II (Yingchuang, Huzhou, China; No. HRP-SD-001/002), and PBS containing 1% BSA; (iv) antigen incubation conditions—37 °C for 0.5 h, 1 h, and 2 h; (v) HRP-conjugated antibody incubation conditions—37 °C for 0.5 h, 37 °C for 1 h, and 2 h. Each parameter was optimized independently while keeping the other parameters constant. The optimal condition for each parameter was selected based on the highest P/N ratio and lowest background signal.

Under the finalized conditions, the capture antibody was coated at 1 μg/mL in PBS (pH 7.4) at 4 °C for 12 h, blocked with commercial microplate stabilizer I at 37 °C for 2 h, the samples were diluted in PBS containing 0.5% Triton X-100, and HRP-conjugated mAb 3F6 was diluted at 1:1000 in commercial HRP stabilizer I. The antigen incubation was performed at 37 °C for 2 h, the HRP-conjugated antibody incubation at 37 °C for 0.5 h, and color development with TMB for 10 min. After stopping the reaction with 2 M H2SO4, absorbance was measured at OD_450_ nm using the VersaMax Microplate Reader (BioTek, Winooski, VT, USA).

### 2.9. Specificity and Repeatability Testing

The specificity of the acELISA was evaluated by testing two rotavirus strains (SD2109 and HB2503) along with 20 anal swabs from healthy horses that were confirmed negative by RT-qPCR, and a panel of common equine pathogens, including EIAV, EIV, EHV-1, EHV-4, EAV, *S. abortus equi*, *E. coli*, and *T. evansi*. Serum-free DMEM was used as the negative control. The cutoff value was defined as the mean OD_450_ of the negative control wells and the 20 anal swabs, plus three times the standard deviation.

The intra-assay precision (repeatability) and inter-assay precision (reproducibility) of the acELISA were assessed using three concentrations of rVP6 protein (high: 50 ng/mL; medium: 10 ng/mL; low: 1 ng/mL). For intra-assay precision, each concentration was tested in eight replicate wells on a single plate in a single run. For inter-assay precision, each concentration was tested in triplicate across three independent experiments. The coefficient of variation (CV%) was calculated as (standard deviation/mean) × 100%. A CV of less than 10% was considered acceptable according to industry standards and the general recommendations of the International Council for Harmonisation (ICH) on the validation of analytical procedures [31].

### 2.10. Standard Curve Establishment

A standard curve was established using two-fold serial dilutions of purified rVP6 protein. The rVP6 protein was diluted in sample diluent (PBS containing 0.5% Triton X-100) to obtain a series of concentrations ranging from 10 to 0.00125 ng/well (equivalent to 100–0.0125 ng/mL at 100 μL/well). Each concentration was tested in triplicate. The OD_450_ values were plotted against the corresponding rVP6 concentrations, and a linear regression equation was derived.

### 2.11. TCID_50_ and RT-qPCR

TCID_50_ assays were performed in Marc-145 cells in 96-well plates using ten-fold serial dilutions with eight replicate wells per dilution. CPE was recorded at 3–4 days post-infection, and titers were calculated by the Reed-Muench method. Based on primers referenced in the literature [32], the RT-qPCR method targeting the NSP3 gene fragment was established: NVP3-F (5’-ACCATCTWCACRTRACCCTC-3’), NVP3-R (5’-GGTCACATAACGCCCCTATA-3’), and NVP3-Probe (5’-ATGAGCACAATAGTTAAAAGCTAACACTGTCAA-3’). Viral RNA was extracted using the TIANamp Virus RNA Kit (TIANGEN, Beijing, China; No. DP315-R) according to the manufacturer’s instructions. RT-qPCR was performed using the HiScript II One Step qRT-PCR Probe Kit (Vazyme, Nanjing, China; No. Q222) in a 25 μL reaction containing 12.5 μL of 2× Probe Mix, 1 μL of Enzyme Mix, 0.5 μL of 50× ROX Reference Dye 1, 1 μM of each primer, 0.5 μM of the probe, 2 μL of RNA template and 6.5 μL of RNase-free ddH_2_O, on a QuantStudio 5 real-time PCR system (Applied Biosystems, Foster City, CA, USA). Cycling conditions were 50 °C for 30 min and 95 °C for 5 min, followed by 40 cycles of 95 °C for 10 s and 60 °C for 30 s, with fluorescence acquired at the end of each 60 °C step. All samples, standards and no-template controls were run in triplicate. A plasmid containing the target sequence was used to establish the standard curve and calculate genome copy numbers.

### 2.12. Ultrafiltration Cutoff Screening and TFF Process Monitoring

To select an ultrafiltration molecular weight cutoff (MWCO), HB2503 harvest was processed with centrifugal polyethersulfone concentrators of 100, 50, or 30 kDa MWCO (Pierce™ Protein Concentrator PES, Thermo Scientific, Waltham, MA, USA; No. UFC910096/UFC905096/UFC903096). Each retentate was concentrated 10-fold, reconstituted to the starting volume with PBS, and analyzed together with the corresponding permeate for VP6 content by acELISA, infectivity by TCID_50_, and total protein by BCA assay. VP6 recovery was calculated as the VP6 mass in the retentate divided by the VP6 mass in the starting material.

For pilot-scale processing, HB2503 harvest was concentrated and diafiltered using a TFF system equipped with a 100 kDa MWCO membrane (Thermo Scientific, Waltham, MA, USA; No. PXB100C50). Feed, initial permeate, post-PBS diafiltration permeate, and final retentate were collected. The final retentate was adjusted to half of the starting volume with PBS. VP6 recovery and fold purification were calculated from VP6 concentration, total protein concentration, and process volume.

### 2.13. Statistical Analysis

Replicate numbers and types are specified in the corresponding figure legends and table footnotes. Technical replicates refer to repeated wells or measurements of the same sample, whereas independent experimental replicates refer to assays performed on separate occasions. Data from replicate measurements are presented as mean ± standard deviation (SD). Single-run experiments were analyzed descriptively. Linear regression analysis was performed using GraphPad Prism software (version 10.4.1). The coefficient of determination (R^2^) was used to assess the goodness of fit of the standard curve and the correlation between different quantification methods. Statistical significance was defined as *p* < 0.05.

## 3. Results

### 3.1. Generation and Characterization of Rabbit Anti-Rotavirus Polyclonal Antibody

**Figure 1:**
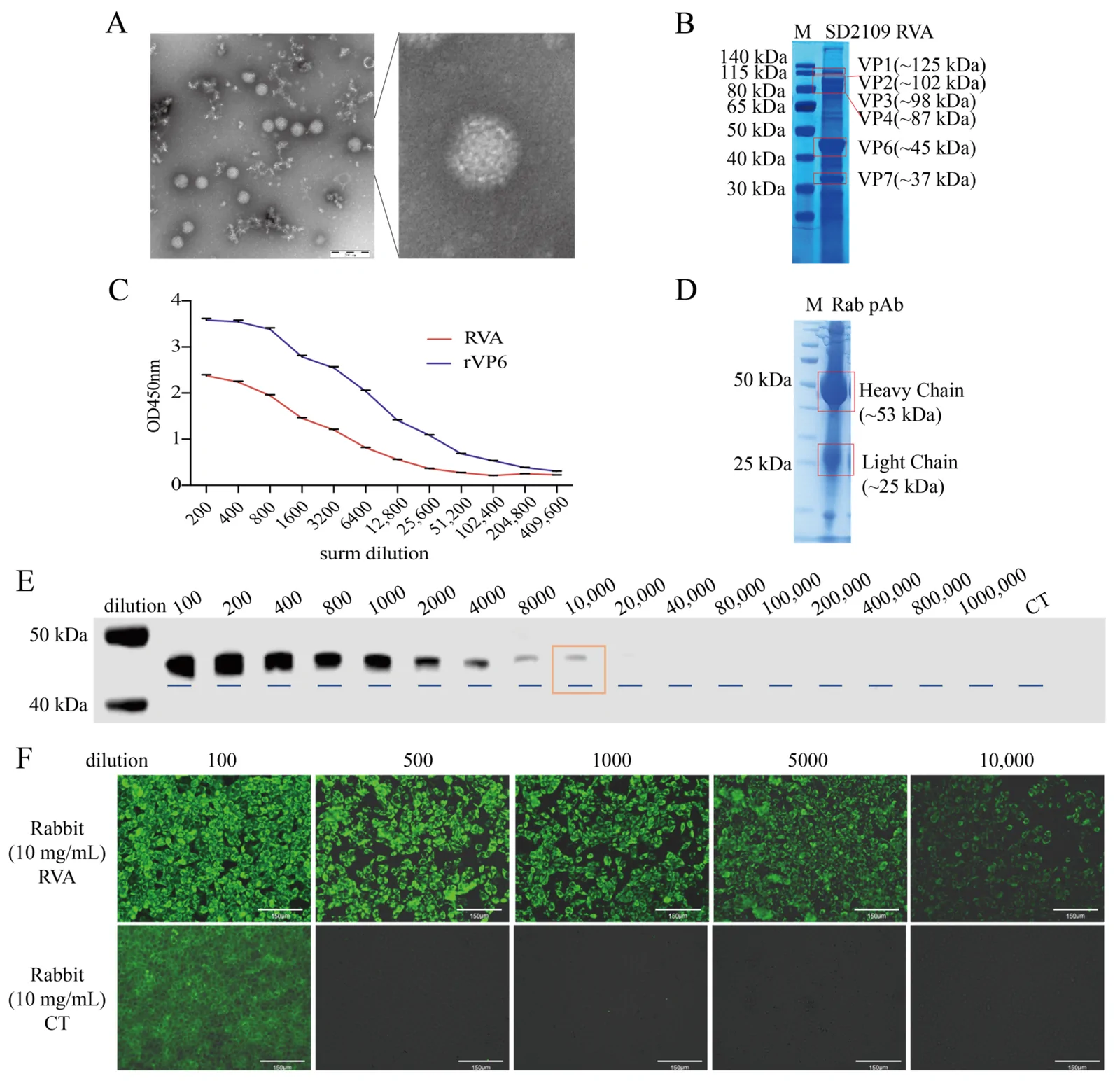
Preparation and characterization of rabbit anti-RVA polyclonal antibody. (**A**) Negative-staining transmission electron microscopy of purified SD2109 particles; scale bar = 200 nm. (**B**) SDS-PAGE of purified virions showing major structural proteins. (**C**) Indirect ELISA titers against purified SD2109 virions and rVP6. Data are presented as mean ± SD of three technical replicated wells, biological replicates *n* = 3. (**D**) SDS-PAGE of purified rabbit IgG. (**E**) WB reactivity of purified rabbit IgG against rVP6 across serial dilutions. (**F**) IFA staining of SD2109-infected Marc-145 cells with purified rabbit IgG; scale bar = 150 μm.

Purified SD2109 virions displayed typical rotavirus-like icosahedral particles of approximately 70–75 nm by negative-staining transmission electron microscopy (Figure 1A). The concentration of the purified virus was determined by measuring total protein content with a spectrophotometer, and the final concentration was adjusted to 2 mg/mL with PBS. SDS-PAGE resolved the expected structural protein profile, including VP1 (~125 kDa), VP2 (~102 kDa), VP3 (~98 kDa), VP4 (~88 kDa), VP6 (~45 kDa), and VP7 (~34 kDa), with VP6 as a prominent band (Figure 1B). After the fourth immunization, the indirect ELISA titers of the antisera against both purified RVA-SD2109 virions and the prokaryotically expressed VP6 protein reached >1:12,800 (Figure 1C). Caprylic acid–ammonium sulfate purification yielded IgG with heavy- and light-chain bands of approximately 50 and 25 kDa, respectively (Figure 1D). The purified pAb recognized VP6 by WB to a dilution of 1:10,000 and strongly stained SD2109-infected Marc-145 cells by IFA (Figure 1E,F). The antibody concentration, as determined by the spectrophotometer, was 10 mg/mL. WB analysis showed an antibody titer of 1:10,000 (Figure 1E), while the indirect immunofluorescence assay against the virus demonstrated a titer of >1:10,000 (Figure 1F).

### 3.2. Generation and Characterization of Mouse Anti-VP6 Monoclonal Antibody

**Figure 2:**
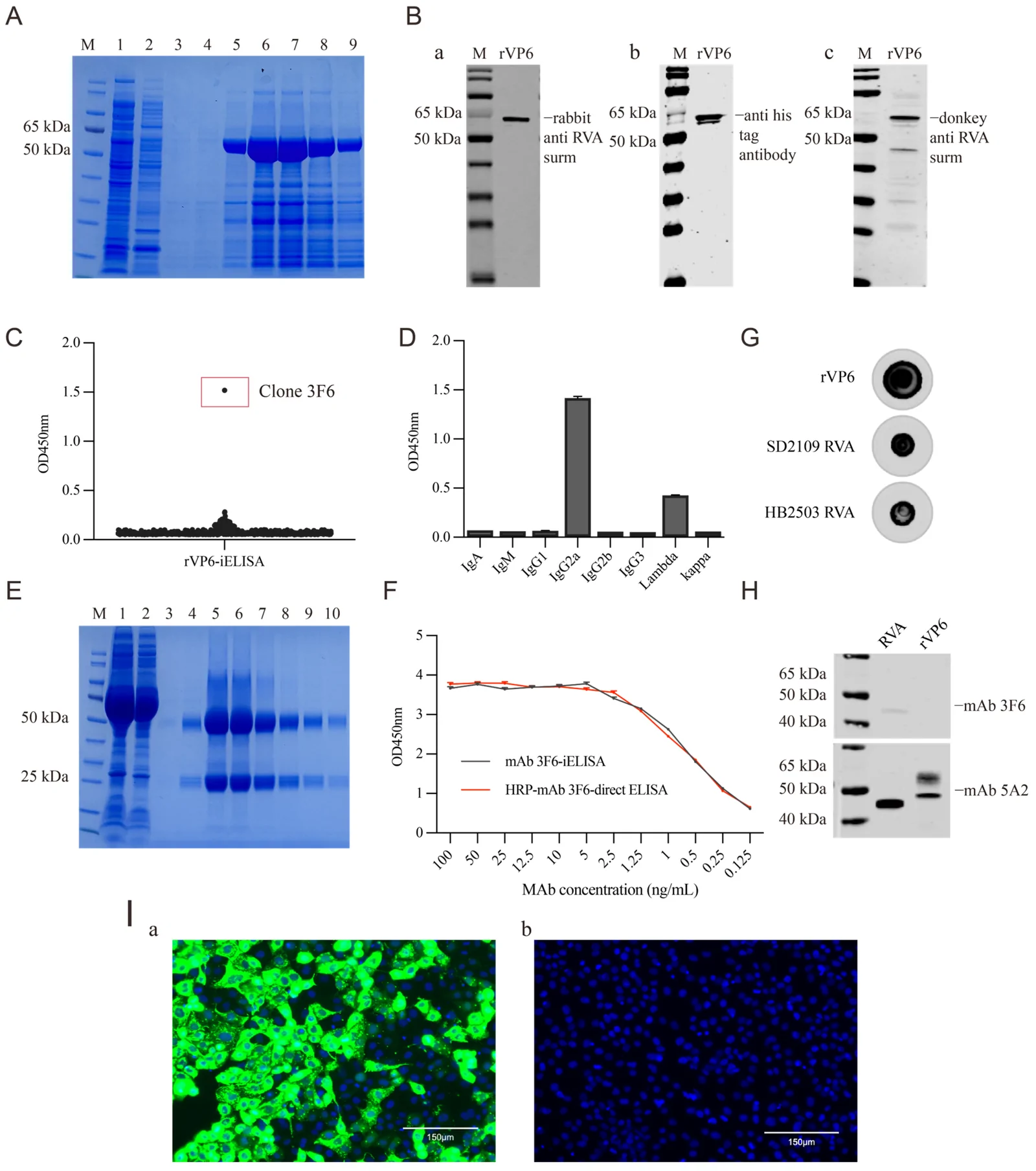
Preparation and characterization of mouse anti-VP6 mAb 3F6. (**A**) SDS-PAGE analysis of Ni-NTA-purified rVP6. Lane 1: clarified cell lysate supernatant; Lane 2: early wash fraction (50 mM imidazole, beginning of wash); Lane 3: late wash fraction (50 mM imidazole, end of wash); Lanes 4–9: elution fractions collected sequentially with 500 mM imidazole. (**B**) WB validation of rVP6 with rabbit anti-RVA serum (**a**), anti-His tag antibody (**b**), and donkey anti-RVA positive serum (**c**). (**C**) Hybridoma screening by indirect ELISA. The primary hybridoma screening was performed once. Candidate positive wells were re-tested during subsequent confirmation rounds. (**D**) Isotype determination. (**E**) SDS-PAGE of purified mAb 3F6. Lane 1: crude mouse ascites; Lanes 2–3: wash fractions; Lanes 4–10: sequential elution fractions. (**F**) Concentration-dependent reactivity of unlabeled and HRP-conjugated mAb 3F6. Data from figures (**D**,**F**) are presented as mean ± SD of three technical replicate wells. (**G**) Dot blot with native rVP6 and virions. (**H**) WB analysis demonstrating that mAb 3F6 recognizes a conformational epitope, whereas mAb 5A2 recognizes a linear epitope. (**I**) IFA staining of mAb 3F6 on Marc-145 cells. (**a**) SD2109 virus-infected cells. (**b**) uninfected negative control cells. Nuclei are counterstained with DAPI (blue); scale bar = 150 μm.

The SD2109 VP6 gene (1194 bp, Figure S1) of the SD2109 strain was cloned into pET-32a(+) using BamHI and Hind III restriction enzymes. The insert sequence was aligned with the parental SD2109 VP6 sequence using SnapGene 8.2.1; no nucleotide substitutions, insertions or deletions were detected, confirming that the cloned ORF was identical to that of the parental strain. The soluble His-tagged rVP6 was obtained most efficiently after induction with 0.5 mmol/L IPTG at 25 °C for 20 h. Ni-NTA purification generated a dominant rVP6 band of approximately 62.6 kDa (Figure 2A), and its identity was confirmed by WB with anti-His antibody, rabbit anti-RVA serum, and donkey anti-RVA positive serum (Figure 2B). Hybridoma screening identified clone 3F6 as the strongest SD2109-reactive clone (OD_450_ > 1.5) (Figure 2C). mAb 3F6 reacted with native rVP6 and native virions in a dot blot but not with denatured rVP6 by WB, indicating recognition of a conformational epitope (Figure 2G,H). It also detected RVA-infected Marc-145 cells by IFA (Figure 2I). The antibody was classified as IgG2a/λ, purified from ascites, and successfully conjugated to HRP while retaining concentration-dependent immunoreactivity (Figure 2D,E,F). The E/P ratio of antibody 3F6 was 2:1, and the final antibody concentration was 0.8 mg/mL.

### 3.3. Optimization of acELISA Conditions

**Figure 3:**
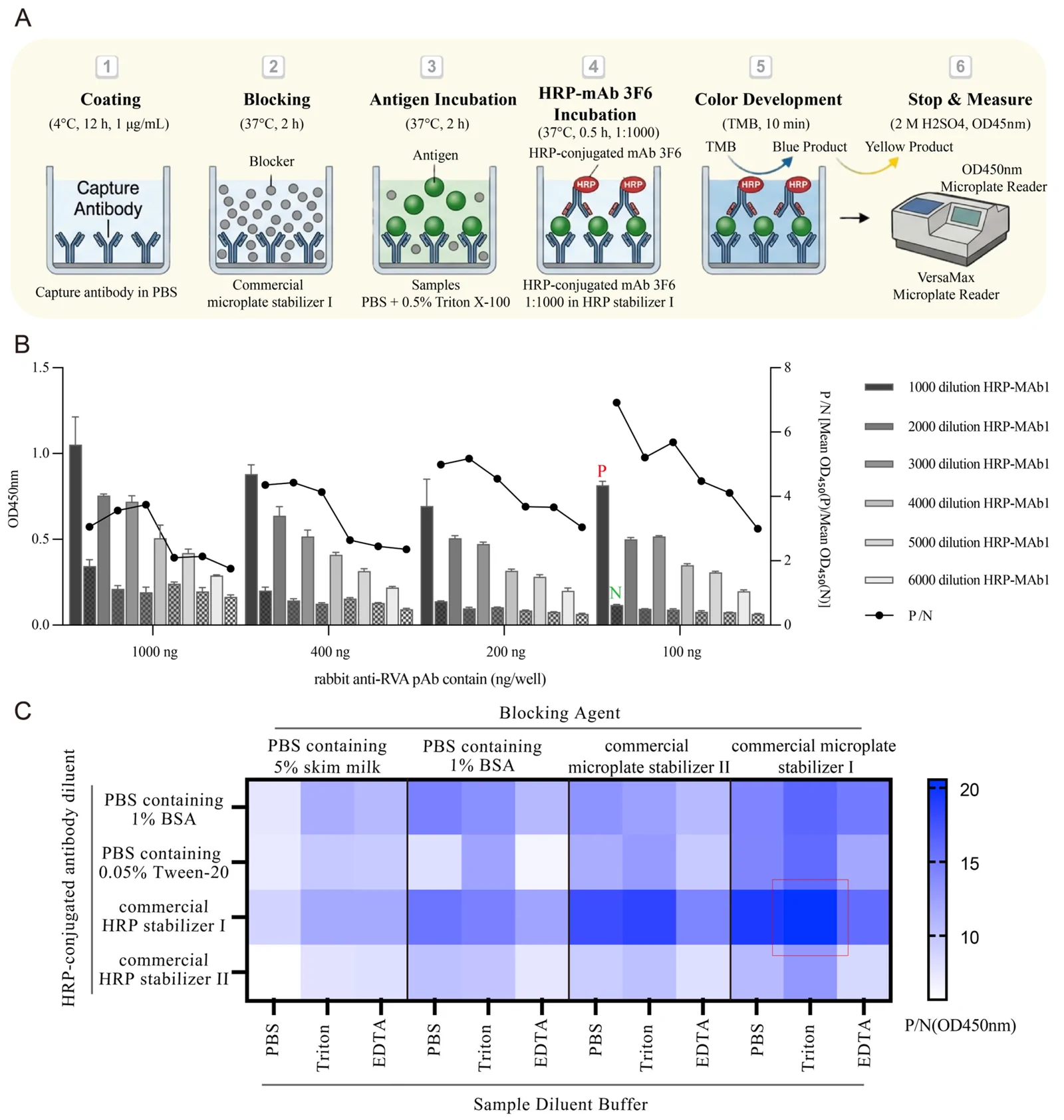
Optimization of the acELISA. (**A**) Schematic diagram of the optimized acELISA workflow. (**B**) Checkerboard titration of capture antibody concentration and HRP-mAb 3F6 dilution. Bar graphs represent OD_450_ values for positive (P) and negative (N) samples at each antibody combination; the line graph represents the corresponding P/N ratio. (**C**) Heatmap of P/N ratios for blocking buffers, sample diluents, and HRP-antibody diluents. The selected condition is indicated by a red box. The optimal combination is highlighted with a red box, yielding the highest P/N ratio (>15). Data of figure (**C**) is from three independent experiments. Data are presented as mean ± SD. The P/N ratio was calculated as the mean OD_450_ of the positive samples divided by the mean OD_450_ of the negative samples.

The optimal acELISA conditions were determined by checkerboard titration analysis performed in three independent experiments. The highest P/N ratio was achieved with a capture polyclonal antibody coating concentration of 1 μg/mL and HRP-conjugated mAb 3F6 diluted at 1:1000 (Figure 3B). Optimization of the key assay parameters identified PBS (pH 7.4) as the optimal coating buffer (coating at 4 °C for 12 h), commercial microplate stabilizer I as the blocking buffer (blocking at 37 °C for 2 h), PBS containing 0.5% Triton X-100 as the sample diluent, and commercial HRP stabilizer I as the diluent for the HRP-conjugated antibody, resulting in the lowest background and the highest P/N ratio (Figure 3C). Further optimization showed that the optimal reaction conditions were antigen incubation at 37 °C for 2 h, HRP-conjugated antibody incubation at 37 °C for 0.5 h, and TMB development for 10 min. Under these conditions, the greatest discrimination between positive and negative samples was achieved (Figure 3A).

### 3.4. Specificity and Repeatability of acELISA

**Figure 4:**
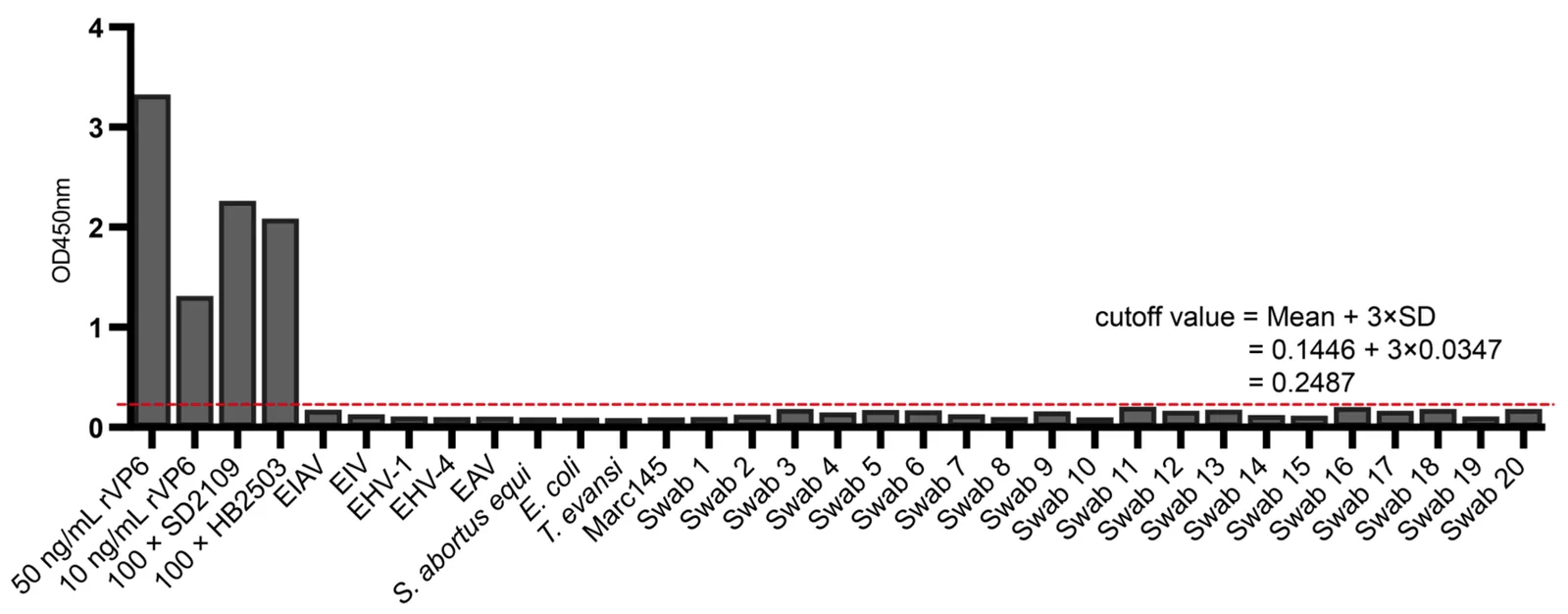
Specificity of the acELISA. OD_450_ values are shown for rVP6 controls, donkey-origin RVA strains SD2109 and HB2503, eight common equine pathogens, uninfected Marc-145 cell lysate, and 20 RT-qPCR-negative anal swabs. The dashed red line indicates the cutoff value of 0.2487. Each sample was analyzed in three technical replicate wells. Data are presented as mean ± SD.

The specificity of the acELISA was assessed by determining its cross-reactivity with common equine enteric pathogens and pathogens responsible for common viral diseases in equids. Both SD2109 and HB2503 strains produced strong positive signals, while no cross-reactivity was observed with EIAV, EIV, EHV-1, EHV-4, EAV, *S. abortus equi*, *E. coli* or *T. evansi,* with OD values comparable to the negative control. These results demonstrated that the acELISA was highly specific for rotavirus detection. The mean of the relative OD_450_ values for the 21 samples was calculated to be 0.1446, and the standard deviation was 0.0347. Therefore, the cutoff value = 0.1446 + 3 × 0.0347 = 0.2487 (Figure 4).

The intra-assay and inter-assay precision results are summarized in Table 1. The intra-assay CV ranged from 2.85% to 5.32%, while the inter-assay CV ranged from 0.77% to 3.16%, both within the acceptable range of < 10%. These results indicated that the acELISA exhibited excellent repeatability and reproducibility.

### 3.5. Standard Curve Based on Recombinant VP6 Protein

**Figure 5:**
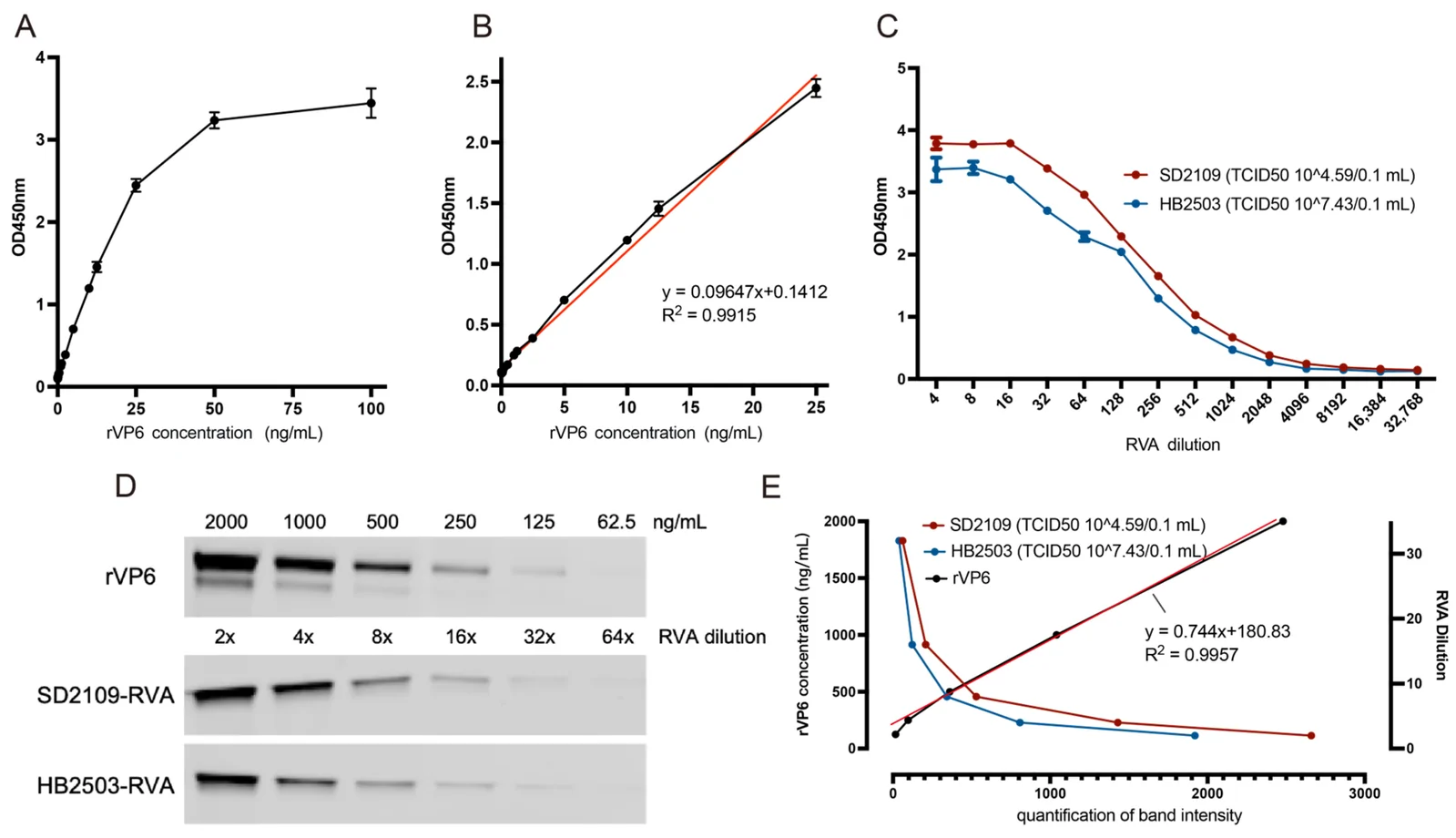
Standard curve and cross-validation of the acELISA. (**A**) Full-range dose-response curve of the acELISA using two-fold serial dilutions of purified rVP6 protein (0.0125–100 ng/mL). OD_450_ values are plotted against rVP6 concentration. Data are presented as mean ± SD (*n* = 3). (**B**) Linear range from 1 to 25 ng/mL rVP6. Linear regression analysis yielded the equation y = 0.09647x + 0.1412 (R^2^ = 0.9915), where y represents OD_450_ and x represents rVP6 concentration (ng/mL). Black data points: Raw experimental measurements used to generate the calibration curve. Red regression line: Quantification of the relationship between Y-axis and X-axis. (**C**) The acELISA OD_450_ values of serial dilutions of two donkey-origin rotavirus strains. (**D**) WB quantification using mAb 5A2 with serially diluted rVP6 and virus samples. Upper panel: two-fold serial dilutions of purified rVP6 protein (2000–62.5 ng/mL); lower panels: two-fold serial dilutions of SD2109 and HB2503 strain (2×–64× dilution). (**E**) Correlation between WB band densitometry (x-axis) and rVP6 concentration or viral dilution (y-axis). Black data points: Raw experimental measurements used to generate the calibration curve. Red regression line: Quantification of the relationship between Y-axis and X-axis. Linear regression of the rVP6 standard: y = 0.744x + 180.83 (R^2^ = 0.9957). Band densities from the rVP6 dilution series were used to construct a standard curve for estimating VP6 content in viral samples.

To establish a quantitative basis for the antigen-capture ELISA (acELISA), a standard curve was created using purified rVP6 protein (Figure 5A). Within the defined linear detection range of 0.1 to 2.5 ng/well (equivalent to 1–25 ng/mL), OD_450_ values exhibited a strong positive correlation with rVP6 concentration. Linear regression analysis yielded the equation: y = 0.09647x + 0.1412 (R^2^ = 0.9915), where y represents the rVP6 concentration (ng/mL) and x represents the corresponding OD_450_ value (Figure 5B). The limit of detection (LOD) was defined as an OD_450_ value above 0.0249, which corresponded to approximately 1 ng/mL under these experimental conditions. The coefficient of determination (R^2^ = 0.9915) indicated an acceptable linear fit across this range, confirming the suitability of the assay for quantitative detection within these boundaries.

### 3.6. Comparison of the acELISA and Other Methods

To compare the quantitative performance of the established acELISA with other commonly used methods, two rotavirus strains were measured against WB analysis, TCID_50_ determination, and quantitative reverse transcription PCR (RT-qPCR). A monoclonal antibody, mAb 5A2, recognizing a conserved linear epitope of VP6 was first used in WB analysis. A standard curve was generated based on the band densitometry of recombinant VP6, and the VP6 content in the viral samples was subsequently estimated (Figure 5D). The acELISA measured 4.77 μg/mL VP6 in SD2109 and 3.32 μg/mL in HB2503, whereas WB estimated 4.82 and 3.54 μg/mL, respectively (Table 2; Figure 5C–E).

Despite comparable VP6 antigen concentrations, SD2109 and HB2503 differed significantly in infectivity. SD2109 had a titer of 4.59 log10 TCID_50_/0.1 mL, whereas HB2503 reached 7.43 log10 TCID_50_/0.1 mL. RT-qPCR detected NSP3 genome copies of 5.79 and 5.09 log10 copies/μL for SD2109 and HB2503, respectively (Table 2). Thus, VP6 antigen content, genome copy number, and infectious titer reflected different properties of the viral preparations, with substantial strain-specific differences in particle-to-infectivity relationships. These comparisons are presented descriptively for the two available strains.

### 3.7. Application of acELISA in Pilot-Scale Vaccine Manufacturing

Small-scale MWCO screening showed that decreasing MWCO improved VP6 retention but reduced filtration efficiency, as lower MWCO prolonged centrifugation time to reach the same final volume. Specifically, to concentrate 15 mL to 1.5 mL (10-fold), the 100, 50, and 30 kDa devices required 10–20, 30–40, and 60–70 min, respectively (including brief inspection pauses). After retentates were reconstituted to the starting volume, VP6 concentrations were 8.477, 12.358, and 14.764 μg/mL for 100, 50, and 30 kDa devices, respectively, compared with 16.782 μg/mL before filtration (Table 3). The corresponding total protein concentrations in retentates were similar (0.531–0.578 mg/mL). Considering throughput, impurity removal, and process scalability, the 100 kDa MWCO membrane was selected for pilot-scale TFF evaluation.

During TFF processing of HB2503, the feed contained 9.360 μg/mL VP6 and 2.333 mg/mL total protein. The initial permeate contained only 0.299 μg/mL VP6 but 2.294 mg/mL total protein, indicating substantial passage of soluble impurities with limited antigen loss. After two PBS diafiltration cycles, the permeate contained 0.058 μg/mL VP6 and 0.032 mg/mL total protein. The final retentate, formulated to half of the starting volume, contained 14.169 μg/mL VP6 and 0.196 mg/mL total protein (Table 4). Overall VP6 recovery was 75.69%, and apparent VP6 purity increased from 0.40% in the feed to 7.23% in the final retentate, representing an approximately 18-fold enrichment.

## 4. Discussion

Rotavirus (RV), a member of the family *Sedoreoviridae* (formerly *Reoviridae*), is a leading causative agent of acute gastroenteritis and diarrheal disease in a wide range of hosts, posing significant threats to both public health and the global livestock industry [3]. Infections have been extensively documented in humans, swine, and cattle, and RV has also been reported in companion and farm animals, including dogs, cats, and equids, highlighting its broad host tropism [33,34]. In the context of animal husbandry, RV-associated diarrhea contributes to substantial economic losses through high morbidity and mortality in neonates, as well as increased veterinary costs and reduced productivity [2]. Of particular concern is the zoonotic potential of equine-like rotaviruses, which harbor reassortant genomic constellations capable of bridging the human–animal interface and thus represent an underappreciated threat to public health security [35,36]. In China, Dong et al. first reported the identification of a G3P[12] reassortant rotavirus strain in donkeys, providing the first molecular characterization of donkey-origin RV in this region in 2022 [7]. The SD2109 strain belongs to the same G3P[12] lineage and is phylogenetically closely related to the sequences reported in that study, with all strains originating from the same locality. More recently, in 2025, large-scale outbreaks of fatal diarrhea among donkey foals were recorded in another province of China, presenting with a markedly accelerated onset and considerably higher case fatality rates compared to previously described cases. Through a systematic virological investigation conducted in our laboratory and subsequent confirmation by the Rotavirus Classification Working Group (RCWG) [37], the causative strain was identified as a novel P genotype, designated G3P[59], the full experimental characterization of which is detailed in our unpublished data (manuscript in preparation). These emerging epidemiological trends suggest that RV infection in equids is spreading and intensifying in China, warranting urgent and heightened veterinary attention. Vaccination represents the most effective intervention strategy for controlling RV-associated disease, and the emergence of a novel genotype such as G3P[59] underscores the pressing need to develop targeted vaccines against this strain [38,39].

This study established a VP6-targeted acELISA for quantitative analysis of donkey-origin RVA and demonstrated its use in vaccine manufacturing process monitoring. The assay addresses a practical gap between infectivity-based assays and nucleic acid-based assays rather than replacing TCID_50_ and RT-qPCR. The acELISA quantifies the content of the intermediate capsid layer (VP6 protein), which does not necessarily correlate with infectivity. Full infectivity requires the presence of an intact outer capsid composed of correctly cleaved VP4 and VP7 proteins, as well as a replication-competent genome. TCID_50_ remains essential for live virus harvest characterization, and RT-qPCR is useful for sensitive genome detection, but neither directly reports antigen mass or antigenic integrity after downstream processing or inactivation [12,13]. By measuring VP6 antigen with an antibody pair that includes a native-epitope-recognizing detector mAb, the acELISA provides a direct, rapid, and plate-based readout suited to in-process decisions.

VP6 was selected because it is abundant, immunogenic, and relatively conserved within RVA, which supports cross-strain detection [14,15]. Structurally, VP6 consists of two distinct domains: a distal beta barrel domain that interacts with the outer capsid and a proximal alpha helical domain that contacts the inner capsid. Its trimeric surface epitopes are conformation-dependent, a feature that directly guided our choice of detector antibody in the antigen-capture assay. In terms of sequence conservation, VP6 is highly conserved among group A rotaviruses, with amino acid sequence identity typically exceeding 90% among strains within the same group. Subgroup antigenic specificity is determined by conformational epitopes formed by VP6 folding or monomer interactions [6,40,41]. The use of polyclonal IgG for capture likely improves antigen recovery by recognizing multiple epitopes, whereas mAb 3F6 provides detector specificity. The inclusion of 0.5% Triton X-100 in the sample diluent may improve access to VP6 epitopes by partially disrupting the outer capsid without abolishing the conformational epitope recognized by 3F6.

The comparison among acELISA, WB, TCID_50_, and RT-qPCR highlights the importance of defining the intended analytical attribute. The numerically comparable results between acELISA and WB suggest that the acELISA could provide reliable VP6 quantification in these samples. In contrast, the nearly three-log difference in infectious titer between SD2109 and HB2503 at similar VP6 concentrations indicates that antigen content cannot be used as a universal surrogate for infectivity. This discrepancy is likely attributable, first and foremost, to the distinct P genotypes of the two strains: SD2109 belongs to G3P[12], whereas HB2503 is a novel G3P[59] strain. VP4, encoded by the P genotype, is the major outer capsid protein responsible for receptor binding, membrane penetration, and viral entry, and it is well established as the primary determinant of cell tropism and specific infectivity [42]. Therefore, sequence divergence in VP4 between the P[12] and P[59] genotypes would be expected to affect entry efficiency, receptor engagement, and proteolytic cleavage susceptibility, all of which directly influence the particle-to-infectivity ratio. This P-genotype-driven difference is very likely the predominant factor underlying the observed three-log gap in infectious titer at comparable VP6 antigen levels. Additional contributing factors may include variations in particle integrity, assembly efficiency, and cell culture adaptation [43,44,45,46,47]. For vaccine manufacturing, this distinction is useful rather than problematic: acELISA is best suited to antigen quantification and recovery monitoring, whereas TCID_50_ and RT-qPCR provide complementary information about infectious virus and genome load before inactivation. We acknowledge that the comparison was performed with only two donor-origin RVA strains currently available in our laboratory, which precludes formal statistical evaluation of method agreement. A larger panel of field isolates will be required to further validate the broader applicability of this acELISA.

The standard curve based on purified rVP6 showed excellent linearity (R^2^ = 0.9915) within the range of 1–25 ng/mL, with a limit of detection of approximately 0.1 ng/well. The intra-assay and inter-assay CVs were within the generally accepted threshold of 10%, indicating robust precision suitable for routine quality control and process monitoring applications. The practical application of the acELISA in monitoring the TFF-based concentration and purification process demonstrated its value as a process analytical technology (PAT) tool. The data showed that the TFF process achieved a VP6 recovery rate of 75.69% and an approximately 18-fold increase in purity, with minimal antigen loss in the permeate fractions. These results are consistent with the expected performance of TFF for virus concentration, and the ability to track VP6 content at each process step enabled rational evaluation and optimization of operating parameters. For instance, the MWCO optimization experiment, guided by acELISA quantification, showed that 30/50 kDa membranes retained more VP6 than the 100 kDa device but required substantially longer processing times (60–70 and 30–40 vs. 10–20 min), while retentate protein levels were similar across all three (0.531–0.578 mg/mL). The improvement in antigen recovery was offset by significantly reduced filtration efficiency and no substantial improvement in impurity removal. Moreover, since free VP6 (~45 kDa) may pass through the 100 kDa membrane, the retained VP6 likely better represents intact virions. Thus, the 100 kDa MWCO was selected as the optimal balance between recovery, purity, and practical feasibility for pilot-scale TFF evaluation. This type of data-driven process optimization would not be possible without a reliable, rapid antigen quantification method such as the acELISA.

High specificity was confirmed by the absence of cross-reactivity with a comprehensive panel of eight common equine and donkey pathogens, including both viral and non-viral agents. However, no clinical validation has been performed in the present study, so the current data only demonstrate analytical specificity and do not establish diagnostic sensitivity or specificity, and the assay should not, at this stage, be described as a validated diagnostic test. Extension of the assay to clinical fecal and anal swab specimens is nevertheless a logical next step, but this poses substantial challenges. Feces contain proteases, bile salts, mucin and inhibitors that can interfere with VP6 detection, so a suitable extraction buffer would need to be confirmed, and a cutoff value would need to be established and validated by ROC analysis against RT-qPCR using a large panel of field samples. Nevertheless, validation with field-derived clinical samples is still in progress, because sample collection on commercial farms requires substantial coordination and has not yet been completed at the scale required for rigorous evaluation. Moreover, because fecal and anal swab specimens generally require dilution prior to testing, and because the amount of material collected at sampling cannot be accurately standardized, these specimen types are less suitable for reliable quantitative analysis. Should qualitative detection be the primary objective, integrating the developed antibodies into lateral flow immunoassay formats, such as colloidal gold or latex particle assays, may offer a more convenient option for point-of-care use in farm settings and could potentially outperform acELISA in terms of field applicability [48,49]. Related studies are currently underway in our laboratory.

## 5. Conclusions

In conclusion, this study has successfully developed and validated a VP6-targeted antigen-capture ELISA for the quantitative detection of donkey-origin group A rotavirus. The successful application of this assay in monitoring antigen recovery during TFF-based vaccine manufacturing underscores its practical value as a process analytical tool, contributing to the rational optimization of production parameters and ensuring batch-to-batch consistency in donkey rotavirus vaccine development.

## Figures and Tables

**Table 1:** Intra-assay and inter-assay precision of the acELISA.

Concentration Level	rVP6 (ng/mL)	Mean OD_450_ ± SD	CV% (Intra-Assay)	CV% (Inter-Assay)
High	50	3.257 ± 0.0957	2.85%	2.49%
Medium	10	1.229 ± 0.0618	5.32%	3.16%
Low	1	0.252 ± 0.0087	3.74%	0.77%

Table note: Intra-assay precision was evaluated using eight technical replicate wells in one run. Inter-assay precision was evaluated in three independent experiments.

**Table 2:** Comparison of VP6 antigen content and viral titers determined by acELISA, Western blot, TCID_50_, and RT-qPCR for two donkey-origin rotavirus strains.

RVA Strain	acELISA (rVP6-μg/mL)	WB (rVP6-μg/mL)	TCID_50_ (log TCID_50_/0.1 mL)	RT-qPCR (NSP3-log Copies/μL)
SD2109	4.77	4.82	4.59	5.79
HB2503	3.32	3.54	7.43	5.09

Table note: acELISA and RT-qPCR were performed with three technical replicate wells; Western blotting was conducted once as a representative result; TCID_50_ was determined using eight replicate wells per dilution. The mean value is reported. Statistical comparison between the two strains was not performed due to the limited number of strains (*n* = 2).

**Table 3:** Comparison of retentate and permeate fractions obtained by centrifugal ultrafiltration devices with different molecular weight cut-offs (MWCOs).

	Time *	Retentate			Permeate		
	(min)	TCID50 (log TCID_50_/0.1 mL)	acELISA (rVP6-μg/mL)	BCA (mg/mL)	TCID50 (log TCID_50_/0.1 mL)	acELISA (rVP6-μg/mL)	BCA (mg/mL)
Pre-filtration	/	8.00	16.782	2.019	/	/	/
100 kDa	10–20 min	6.80	8.477	0.531	3.29	1.929	1.528
50 kDa	30–40 min	7.43	12.358	0.551	2.00	0.390	1.506
30 kDa	60–70 min	7.43	14.764	0.578	1.57	0.029	1.578

* Time (min): required to concentrate the same starting volume (15 mL) to a 10-fold reduction (final volume 1.5 mL) using centrifugal ultrafiltration devices with the indicated MWCOs. The ultrafiltration screening was performed once. TCID_50_ titers were measured with eight replicate wells per dilution. acELISA and BCA were conducted once.

**Table 4:** Monitoring of VP6 antigen content and total protein concentration during tangential flow filtration (TFF)-based vaccine manufacturing of the HB2503 rotavirus strain.

	TCID50 (log TCID50/0.1 mL)	acELISA (rVP6-μg/mL)	BCA (mg/mL)
Pre-filtration	7.67 ± 0.00	9.360 ± 0.012	2.333 ± 0.120
Initial permeate	/	0.299 ± 0.003	2.294 ± 0.087
Post-PBS diafiltration permeate	/	0.058 ± 0.000	0.032 ± 0.010
Final product (retentate)	/	14.169 ± 0.177	0.196 ± 0.078

Table note: Data were obtained from one TFF purification process. Each collected sample was analyzed in three independent analytical measurements, and the results are presented as mean ± SD.

## Data Availability

The original contributions presented in this study are included in the article. Further inquiries can be directed to the corresponding author.

## Supplementary Materials

The following supporting information can be downloaded at: https://www.mdpi.com/article/10.3390/microorganisms14091874/s1, Figure S1. PCR amplification of the rotavirus VP6 gene; Figure S2. Schematic map of the recombinant expression plasmid pET-32a-ERVA-VP6; Figure S3. Schematic workflow for the propagation and purification of the SD2109 rotavirus strain and the preparation of rabbit anti-rotavirus polyclonal antibodies; Figure S4. Schematic workflow for the propagation and purification of the SD2109 rotavirus strain and the preparation of rabbit anti-rotavirus polyclonal antibodies.

## Abbreviations

The following abbreviations are used in this manuscript:

RVA	rotavirus A
RCWG	Rotavirus Classification Working Group
acELISA	antigen-capture enzyme-linked immunosorbent assay
iELISA	indirect enzyme-linked immunosorbent assay
IFA	indirect immunofluorescence assay
RT-qPCR	quantitative reverse transcription polymerase chain reaction
SDS-PAGE	sodium dodecyl sulfate polyacrylamide gel electrophoresis
TCID_50_	50% tissue culture infectious dose
TFF	tangential flow filtration
WB	Western blot
BSA	bovine serum albumin
DAPI	4′,6-diamidino-2-phenylindole
DMEM	Dulbecco’s modified Eagle’s medium
FBS	fetal bovine serum
FITC	fluorescein isothiocyanate
HAT	hypoxanthine–aminopterin–thymidine (hybridoma selection medium)
HRP	horseradish peroxidase
IPTG	isopropyl-β-D-thiogalactopyranoside
mAb	mouse monoclonal antibody
Ni-NTA	nickel–nitrilotriacetic acid affinity resin
pAb	polyclonal antibodies
PBST	phosphate-buffered saline containing Tween-20
PEG	polyethylene glycol
TBST	Tris-buffered saline containing Tween-20
TMB	3,3′,5,5′-tetramethylbenzidine
CPE	cytopathic effect
kDa	kilodalton
LOD	limit of detection
MOI	multiplicity of infection
MWCO	molecular weight cut-off
ORF	open reading frame
P/N ratio	ratio of the OD_450_ of the positive sample to that of the negative sample
